# The impact of sleep health on cardiovascular and all-cause mortality in the general population

**DOI:** 10.1038/s41598-025-15828-6

**Published:** 2025-08-16

**Authors:** Soo Jung Park, Jinsun Park, Byung Sik Kim, Jin-Kyu Park

**Affiliations:** 1 Division of Cardiology, Department of Internal Medicine, Hanyang University Medical Center, 222 Wangsimni-ro, Seongdong-gu, Seoul, 04763 Republic of Korea; 2 Division of Cardiology, Hanyang University Guri Hospital, Guri, Republic of Korea

**Keywords:** Sleep, Sleep deprivation, Mortality, Cardiovascular disease, Risk factors, Cardiovascular biology, Health care

## Abstract

Sleep patterns are important predictors of long-term health outcomes. We investigated the associations of sleep duration, sleep regularity, and their combinations with all-cause mortality and cardiovascular events in a large Korean cohort, including sex-specific analyses. This prospective cohort study included 9,641 adults aged 40–69 years from the Ansung–Ansan cohort in Korea. Participants were categorized by self-reported sleep duration (< 7 h, 7–8 h, or > 8 h) and regularity (regular vs. irregular). Outcomes included all-cause mortality and major adverse cardiovascular events (MACE) over a median follow-up of 186 months, during which 1,095 deaths and 811 MACE occurred. Sleep duration > 8 h was associated with increased all-cause mortality (adjusted hazard ratio [HR], 1.27; 95% confidence interval [CI], 1.04–1.54). Combined analysis showed higher mortality in those with < 7 h and irregular sleep (adjusted HR, 1.28; 95% CI, 1.04–1.58) and > 8 h and regular sleep (adjusted HR, 1.26; 95% CI, 1.01–1.58). In sex-specific analyses, sleep duration and regularity were differentially associated with mortality. In women, > 8 h of irregular sleep was associated with increased risk, while in men, < 7 h of irregular or > 8 h of regular sleep showed similar associations. These findings support tailored strategies considering both sleep characteristics and sex.

**Supplementary Information:**

The online version contains supplementary material available at 10.1038/s41598-025-15828-6.

## Introduction

Sleep is an essential activity in human life that influences inflammation^1,2^ and metabolic profiles^3^ which are risk factors for cardiovascular diseases and mortality^4,5^. Based on these relationships with intermediate endpoints and biomarkers, sleep is likely to be associated with cardiovascular diseases and mortality.

The concept of sleep health encompasses multiple dimensions such as sleep duration, quality, consistency, and circadian alignment, all of which contribute to physiological and psychological homeostasis. Disruptions in these aspects have been associated with an increased risk of hypertension^6^ atherosclerosis^7^ and metabolic disorders^3^ which collectively increase the risk of cardiovascular and all-cause mortality. Several studies show the relationship between sleep duration and mortality^8,9^. However, most focused exclusively on duration and the findings have been inconsistent, with the shape of the association remaining unclear. Some studies reported a U-shaped relationship between all-cause and disease-specific mortality^10^ showing increased mortality with both < 7 and > 8 h/day of sleep^11^ whereas others found a positive association with prolonged sleep only^12^. These discrepancies likely reflect the complex, multifactorial nature of sleep. Moreover, recent research suggests that the association between sleep duration and adverse outcomes may differ according to sex, potentially due to physiological and behavioral differences^12^.

Beyond sleep duration, there has been growing attention paid to sleep quality and its impact on long-term health outcomes. In particular, sleep regularity, defined as the consistency of sleep–wake timing, has emerged as a potentially stronger predictor of cardiometabolic risk than sleep duration alone^13^. Sleep quality is also known to differ between men and women^14^. Given the known sex-based differences in sleep and cardiovascular risk profiles, it is important to assess whether associations between sleep patterns and outcomes differ by sex to inform personalized prevention strategies. However, studies examining the combined effects of sleep duration and quality, along with their sex-specific associations with clinical outcomes, such as mortality and cardiovascular diseases, remain limited.

We aimed to investigate the associations of sleep duration, regularity, and perceived sufficiency with all-cause mortality and cardiovascular events in a large, well-characterized community-based cohort in Korea. We further explored the combined effects of sleep patterns and performed sex-specific analyses to assess the potential sex-based differences.

## Methods

### Study population

This study included 9,641 participants, aged 40–69 years, from the Ansung–Ansan cohort of the Korean Genome Epidemiology Study conducted by the Korea Disease Control and Prevention Agency. This study investigated the genetic and environmental factors contributing to the prevalence of metabolic and cardiovascular diseases. Individuals residing in rural (Ansung) and urban (Ansan) communities were enrolled between June 2001 and January 2003. Detailed protocols have been described previously^15^.

**Fig. 1:**
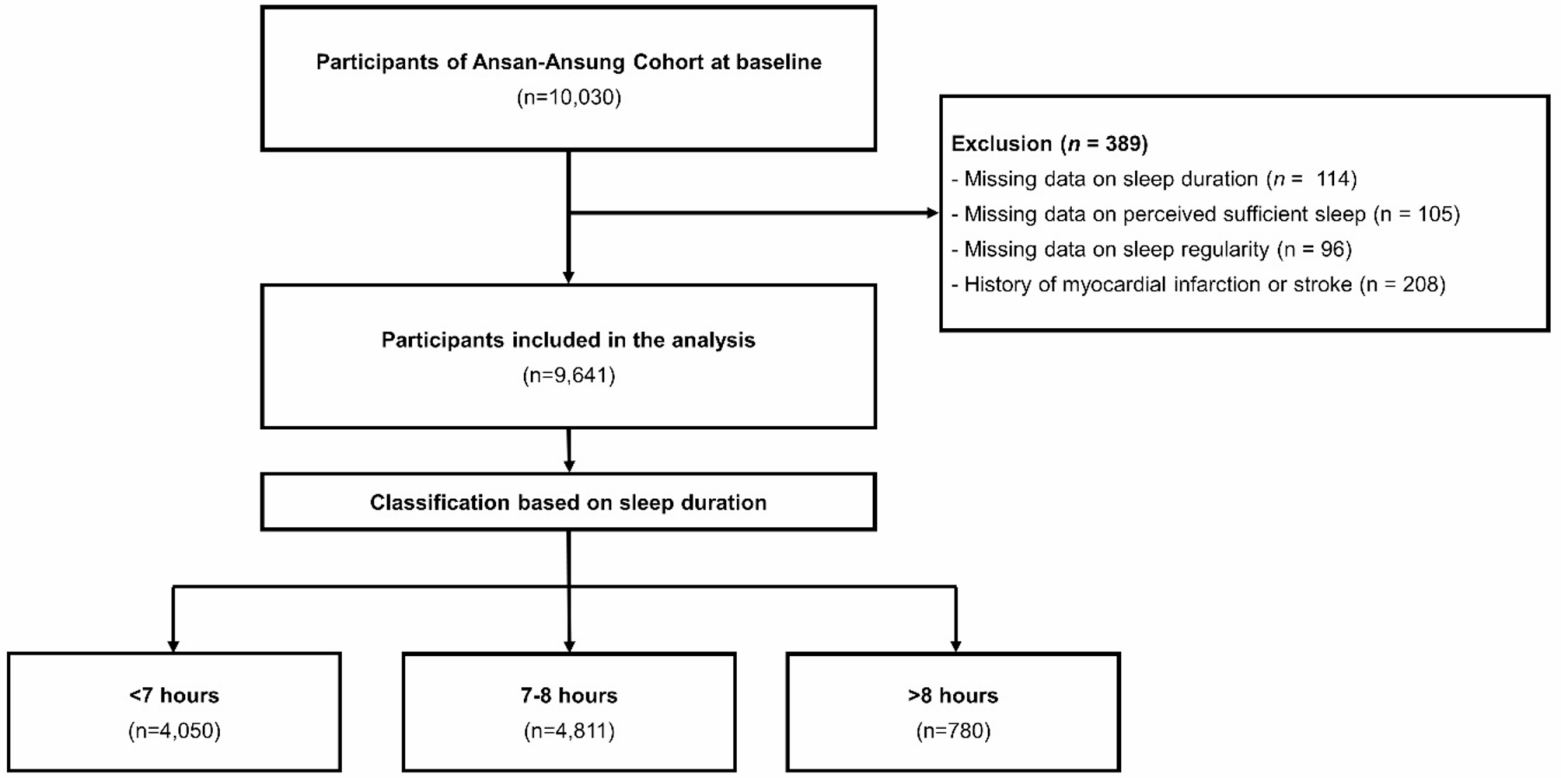
Flow diagram of the study.

A total of 10,030 eligible individuals who had resided in Ansung (*n* = 5,018) or Ansan (*n* = 5,012) for at least 6 months were enrolled in the study. Participants diagnosed with a history of myocardial infarction or stroke at baseline and those without data on sleep duration, sleep regularity, or perceived sufficient sleep were excluded (*n* = 389), resulting in a final population of 9,641 participants. Participants were further categorized into three groups based on self-reported sleep duration: <7 h (*n* = 4,050), 7–8 h (*n* = 4,811), and > 8 h (*n* = 780) (Fig. 1).

Myriad comprehensive health examinations, detailed on-site interviews, and meticulous laboratory tests were conducted during the baseline visit to a tertiary hospital. Eight serial reassessments following the entire cohort protocol were performed through scheduled revisits every other year until 2018. All participants voluntarily enrolled in the study and provided written informed consent at baseline assessment and at each follow-up visit. This study adhered to the principles of the Declaration of Helsinki and was approved by the Korean National Research Institute of Health and the Institutional Review Board of Hanyang University Medical Center (HYUH 2017-12-033).

### Assessment of lifestyle and medical history, physical examination, and laboratory tests

Well-trained investigators conducted comprehensive onsite interviews, collected critical lifestyle and clinical data, and performed physical examinations at tertiary hospitals during each visit. A structured questionnaire was used to obtain data on smoking, alcohol intake, monthly household income, education level, and specific medical conditions, such as hypertension, diabetes mellitus, dyslipidemia, stroke, coronary artery disease, and heart failure. The type and duration of physical activity were assessed using detailed questionnaires and quantified using estimated weekly metabolic equivalent task scores. Trained examiners measured blood pressure using a mercury sphygmomanometer positioned at heart level. Measurements were performed at least twice with the participant in a sitting position, and the results were averaged. If a blood pressure difference of ≥ 5 mmHg was observed between the two measurements, a third measurement was taken, and the last two measurements were averaged. Waist circumference was measured at the midpoint between the lowest rib and iliac crest at the end of expiration in the standing position.

Blood samples were collected after overnight fasting and analyzed to determine lipid profiles, fasting glucose and hemoglobin A1c levels, and serum creatinine levels using an automated analyzer. Laboratory evaluations were performed in a single-core clinical laboratory accredited and participated annually in inspections and surveys conducted by the Korean Association of Quality Assurance for Clinical Laboratories. Blood glucose, total cholesterol, high-density lipoprotein (HDL)-cholesterol, and triglyceride concentrations were measured using the enzyme method (ADVIA 1650 and ADVIA 1800; Siemens Healthineers). Low-density lipoprotein (LDL)-cholesterol levels were calculated using Martin’s equation^16^. The Chronic Kidney Disease Epidemiology Collaboration was used to calculate the estimated glomerular filtration rate (eGFR)^17^. Chronic kidney disease was defined as an eGFR of < 60 mL/min/1.73 m² according to the National Kidney Foundation KDOQI guidelines for chronic kidney disease^18^. Hypertension was defined as a diagnosis of hypertension by a physician, regular use of antihypertensive medications, or systolic blood pressure ≥ 140 mmHg or diastolic blood pressure ≥ 90 mmHg^19^. Diabetes was defined as a diagnosis of diabetes by a physician, regular use of antidiabetic medications, hemoglobin A1c level of ≥ 6.5%, or fasting blood glucose level of ≥ 126 mg/dL^20^. Dyslipidemia was defined as a diagnosis of dyslipidemia by a physician, the regular use of lipid-lowering medications, or the presence of at least one of the following abnormal laboratory test results: total cholesterol level of ≥ 240 mg/dL, LDL cholesterol level of ≥ 160 mg/dL, triglyceride (TG) level of ≥ 200 mg/dL, or HDL cholesterol level of < 40 mg/dL in men or < 50 mg/dL in women^21^.

### Assessment of sleep patterns

Sleep patterns, including duration and quality (regularity and perceived sufficiency), were assessed using a structured questionnaire at the baseline visit. The participants were asked the following questions:

(1) “On average, how many hours of sleep do you typically obtain per night?”

(2) “Do you maintain regularity in your bedtimes and wake-up times?”

(3) “Do you perceive your sleep duration as sufficient?”

The sleep duration was recorded as a continuous variable (in hours). For the analysis, the data were categorized into three groups: <7 h, 7–8 h (reference category), and > 8 h. This categorization was based on prior epidemiological studies that identified both short and long sleep durations (typically defined as < 7 and > 8–9 h, respectively) as associated with increased health risks^22^. Sleep regularity and perceived sufficiency of sleep were assessed as binary variables (Yes/No). For interaction and combined effect analyses, participants were further stratified into six subgroups according to combinations of sleep duration and sleep regularity, and separately with perceived sufficient sleep. The distribution of sleep duration among study participants is presented in Supplementary Fig. 1.

### Outcome definitions and identification

The primary outcome was all-cause mortality, and the secondary outcome was major adverse cardiovascular events (MACEs), defined as a composite of cardiovascular mortality, MI, and stroke. All-cause and cardiovascular mortalities were identified using data from the Korean National Statistics Office, which records the causes of death using the International Classification of Diseases, 10th revision codes. Cardiovascular mortality was defined as death caused by diseases coded as I20–I82, including ischemic heart disease (I21–I25), heart failure (I50), fatal arrhythmia (I46), cerebrovascular events such as ischemic and hemorrhagic stroke (I60–I69), pulmonary thromboembolism (I26–I28), peripheral vascular disease (I70–I75), and sudden cardiac arrest (R96). Newly developed MI and stroke were identified through biennial on-site interviews using a structured questionnaire. MI was defined as a clinical emergency, in which the patient recalled experiencing a heart attack episode necessitating hospitalization or coronary revascularization. Stroke was defined as a clinical emergency in which the patient recalled experiencing a sudden neurological deficit, such as paralysis or language impairment, requiring hospitalization.

### Statistical analysis

Continuous variables are presented as means and standard deviations, and categorical variables as frequencies and percentages. The distribution of continuous variables was assessed using the Kolmogorov–Smirnov test and visually inspected using Q–Q plots. Group comparisons were conducted using one-way analysis of variance followed by Tukey’s post hoc test for normally distributed variables, or the Kruskal–Wallis test followed by Dunn’s multiple comparison test for non-normally distributed variables. Categorical variables were compared using the chi-squared or Fisher’s exact test, as appropriate. The Kaplan–Meier survival analysis with the log-rank test was used to compare the cumulative incidence of primary and secondary outcomes across the three sleep duration groups (< 7 h, 7–8 h, and > 8 h). Cox proportional hazard regression models were used to estimate the hazard ratios (HRs) and 95% confidence intervals (CIs) for the associations between sleep duration, sleep regularity, and perceived sufficient sleep and the outcomes. The 7–8 h group was used as the reference category. Multivariable models were adjusted for clinically relevant covariates, including age (per 10 years), sex, body mass index (BMI), smoking status, alcohol intake, physical activity, hypertension, diabetes, dyslipidemia, eGFR (per 10 mL/min/1.73 m²), area of residence (urban or rural), monthly household income, and education level. To assess the combined effects, participants were further classified into six groups according to combinations of sleep duration, with either sleep regularity (regular vs. irregular) or perceived sufficient sleep (sufficient vs. insufficient). The reference group in both combined analyses was defined as participants reporting 7–8 h of regular or sufficient sleep. Subgroup analyses were conducted separately for men and women to explore the potential sex-specific associations. The proportional hazard assumption was assessed using scaled Schoenfeld residuals. Variance inflation factors (VIFs) were calculated for the variables in the multivariate models. All VIF values were below 2, indicating that multicollinearity was not a concern.

We also compared baseline characteristics between participants who were lost to follow-up before the final survey and those who completed follow-up, to assess potential selection bias. The results are presented in Supplementary Table 1. All statistical analyses were conducted using the R software (version 4.4.3; www.R-project.org) and RStudio (version 2024.12.0; www.rstudio.com). Differences were considered statistically significant at *p* < 0.05 for all analyses. Statistical packages, including tableone, survminer, rms, ggplot2, and survival, were used to perform analyses and generate visualizations.

## Results

### Baseline characteristics

The study population comprised 9,641 individuals (mean age, 52.11 ± 8.89 years; 47.4% men). The baseline characteristics stratified by sleep duration are presented in Table 1. Participants with longer sleep duration (> 8 h) were older (mean age, 55.68 ± 9.10 years), more likely to reside in rural areas (72.8%), and had lower levels of education and income (*p* < 0.001 for all). They also exhibited a higher waist circumference, systolic blood pressure, diastolic blood pressure, and a greater prevalence of hypertension and diabetes than the other groups (all *p* < 0.001). The proportion of men was slightly lower in the short (< 7 h, 44.1%) and long (> 8 h, 46.7%) sleep groups than in the 7–8 h group (50.3%, *p* < 0.001). Laboratory parameters such as HbA1c and triglyceride levels were also less favorable in the long sleep group. In contrast, individuals with short sleep durations (< 7 h) were more likely to live in urban areas, have a higher socioeconomic status, and be current smokers and drinkers, but reported lower levels of physical activity (*p* < 0.001). Sleep regularity and perceived sufficiency differed significantly between the groups. The 7–8 h group had the highest proportion of both regular (75.5%) and sufficient (74.8%) sleep. The lowest prevalence of sufficient sleep was observed in the < 7 h group (49.3%), whereas the lowest prevalence of regular sleep was observed in the > 8 h group (71.2%) (*p* < 0.001 and *p* = 0.032, respectively).

In addition, baseline sleep characteristics differed by sex, with men reporting a slightly longer average sleep duration and a higher prevalence of sufficient sleep, while women were more likely to report sleep durations < 7 h (Supplementary Table 1).

**Table 1:** Baseline characteristics of the study population according to sleep duration.

Characteristic	Overall (*N* = 9,641)	Sleep duration			*P*-value
		< 7 h (*N* = 4,050)	7–8 h (*N* = 4,811)	> 8 h (*N* = 780)	
Age, mean (SD), years	52.11 ± 8.89	51.38 ± 8.72	52.14 ± 8.86^a^	55.68 ± 9.10^ab^	< 0.001
Male sex, N (%)	4571 (47.4)	1785 (44.1)	2422 (50.3)	364 (46.7)	< 0.001
Area					< 0.001
Urban (Ansan)	4881 (50.6)	2513 (62.0)	2156 (44.8)	212 (27.2)	
Rural (Ansung)	4760 (49.4)	1537 (38.0)	2655 (55.2)	568 (72.8)	
Body mass index, N (%), kg/m^2^					0.067
<18.5	170 (1.8)	76 (1.9)	78 (1.6)	16 (2.1)	
18.5–22.9	2772 (29.0)	1128 (28.0)	1399 (29.3)	245 (31.7)	
23.0–24.9	2512 (26.3)	1026 (25.5)	1269 (26.6)	217 (28.1)	
25.0–29.9	3634 (38.0)	1574 (39.1)	1801 (37.7)	259 (33.5)	
≥ 30.0	481 (5.0)	219 (5.4)	227 (4.8)	35 (4.5)	
Waist circumference, mean (SD), cm	82.92 ± 8.79	82.44 ± 8.97	83.19 ± 8.64^a^	83.82 ± 8.65^a^	< 0.001
Monthly income, N (%), *10^4^ KRW					< 0.001
Low (< 100)	3345 (35.2)	1209 (30.2)	1735 (36.7)	401 (52.4)	
Medium (100–199)	2783 (29.3)	1189 (29.7)	1390 (29.4)	204 (26.7)	
High (≥ 200)	3367 (35.5)	1600 (40.0)	1607 (34.0)	160 (20.9)	
Education, N (%)					< 0.001
Lower than middle school	3175 (33.1)	1143 (28.4)	1645 (34.4)	387 (50.1)	
Middle school	2213 (23.1)	907 (22.5)	1134 (23.7)	172 (22.3)	
High school	2923 (30.5)	1342 (33.3)	1407 (29.4)	174 (22.5)	
University and college	1277 (13.3)	639 (15.9)	598 (12.5)	40 (5.2)	
Smoking status, N (%)					< 0.001
Current smoker	2454 (25.7)	922 (23.0)	1311 (27.5)	221 (28.5)	
Ex-smoker	1475 (15.5)	608 (15.2)	747 (15.7)	120 (15.5)	
Non-smoker	5614 (58.8)	2475 (61.8)	2705 (56.8)	434 (56.0)	
Alcohol drinking, N (%)					0.002
Current drinker	4540 (47.4)	1856 (46.1)	2329 (48.7)	355 (45.9)	
Ex-drinker	625 (6.5)	261 (6.5)	291 (6.1)	73 (9.4)	
Non-drinker	4419 (46.1)	1908 (47.4)	2165 (45.2)	346 (44.7)	
Physical activity, N (%), METs-hour/week					< 0.001
<7.5	747 (7.7)	279 (6.9)	375 (7.8)	93 (11.9)	
7.5–14.9	1815 (18.8)	805 (19.9)	876 (18.2)	134 (17.2)	
15.0–30.0	2910 (30.2)	1319 (32.6)	1403 (29.2)	188 (24.1)	
≥30.0	4169 (43.2)	1647 (40.7)	2157 (44.8)	365 (46.8)	
Systolic blood pressure, mean (SD), mmHg	124.51 ± 18.81	123.60 ± 18.89	124.63 ± 18.46^a^	128.45 ± 19.93^ab^	< 0.001
Diastolic blood pressure, mean (SD), mmHg	81.65 ± 11.79	81.03 ± 11.89	81.92 ± 11.67^a^	83.26 ± 11.80^ab^	< 0.001
Medical history, N (%)					
Hypertension	3588 (37.2)	1436 (35.5)	1802 (37.5)	350 (44.9)	< 0.001
Diabetes mellitus	1060 (11.1)	425 (10.5)	517 (10.8)	118 (15.4)	< 0.001
Dyslipidemia	3989 (41.5)	1646 (40.7)	2006 (41.8)	337 (43.3)	0.328
Chronic kidney disease	199 (2.1)	71 (1.8)	108 (2.2)	20 (2.6)	0.158
Coronary artery disease	70 (0.7)	31 (0.8)	32 (0.7)	7 (0.9)	0.722
Heart failure	17 (0.2)	8 (0.2)	8 (0.2)	1 (0.1)	0.89
Laboratory data, mean (SD)					
eGFR, mL/min/1.73m2	92.11 ± 14.26	92.39 ± 14.14	92.02 ± 14.40	91.23 ± 13.93	0.092
Fasting glucose, mg/dL	92.40 ± 22.72	92.29 ± 22.33	92.39 ± 23.06	93.07 ± 22.71	0.108^c^
Hemoglobin a1c, %	5.79 ± 0.93	5.78 ± 0.93	5.78 ± 0.90	5.93 ± 1.08 ab	< 0.001^c^
Total cholesterol, mg/dL	198.52 ± 36.78	199.13 ± 36.69	198.05 ± 36.95	198.28 ± 36.21	0.38
Triglyceride, mg/dL	152.95 ± 109.40	147.71 ± 99.66	157.59 ± 119.69^a^	151.51 ± 88.04^a^	< 0.001^c^
HDL cholesterol, mg/dL	49.57 ± 11.86	50.04 ± 11.88	49.24 ± 11.82^a^	49.22 ± 12.02	0.005
LDL cholesterol, mg/dL	122.35 ± 32.02	123.04 ± 32.17	121.72 ± 31.97	122.61 ± 31.49	0.147
Self-reported sleep quality, N (%)					
Sufficient sleep duration	6235 (64.7)	1998 (49.3)	3601 (74.8)	636 (81.5)	< 0.001
Regular sleep time	7225 (74.9)	3036 (75.0)	3634 (75.5)	555 (71.2)	0.032

Values are presented as mean ± standard deviation or n (%) unless indicated otherwise. The percentages may not total to 100% owing to rounding.

Glomerular filtration rate was calculated using CKD-EPI equations.

^a^Post hoc p: Statistically significant difference at *p* < 0.05, compared with the < 6 h group.

^b^Post hoc p: Statistically significant difference at *p* < 0.05, compared with the 6–8 h group.

^c^Assessed using nonparametric test.

eGFR, estimated glomerular filtration rate; HDL, high-density lipoprotein; KRW, Korean Won; LDL, low-density lipoprotein; METs, metabolic equivalents; SD, standard deviation.

### Associations between sleep patterns with clinical outcomes

**Fig. 2:**
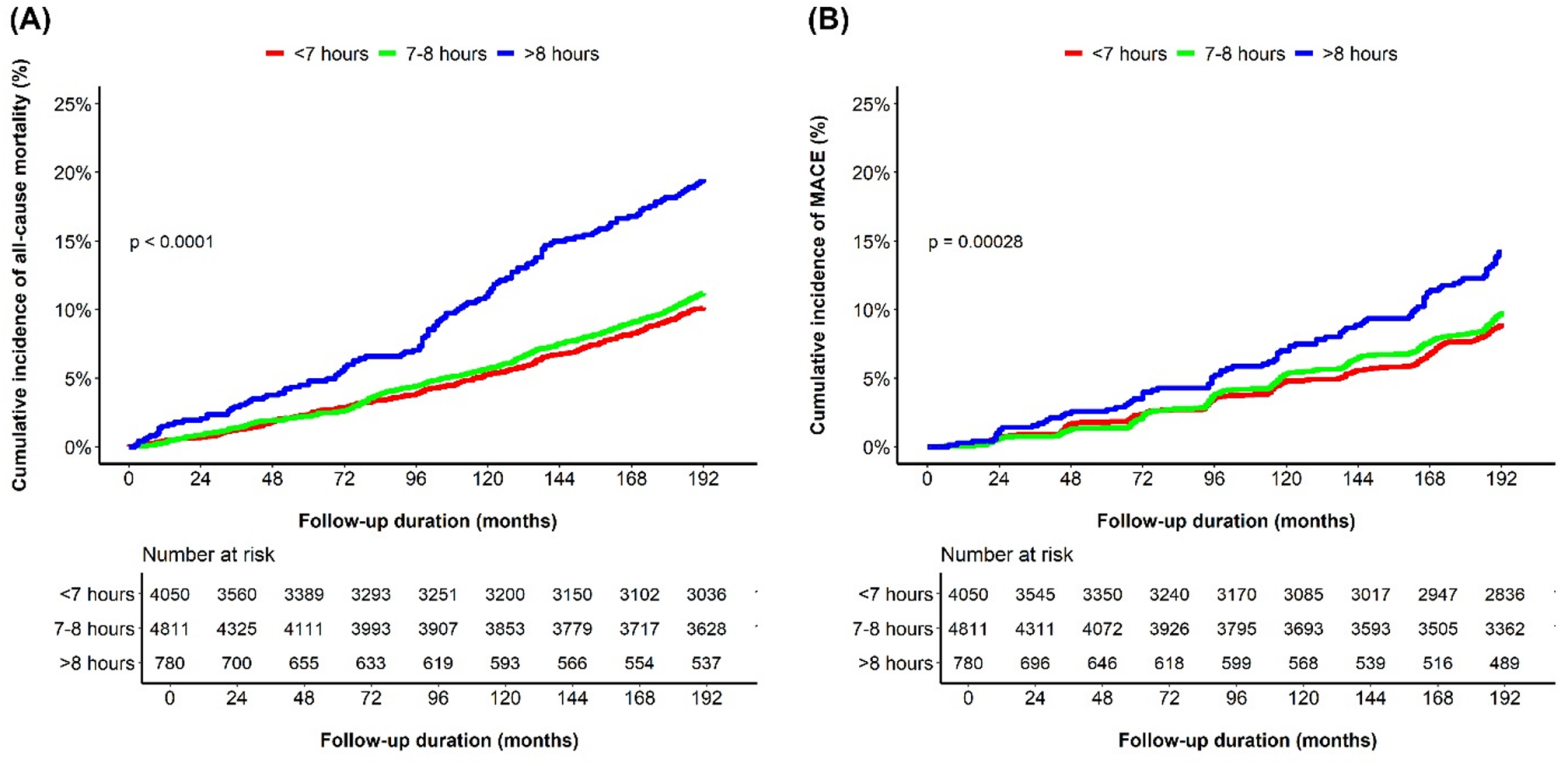
Cumulative incidence of all-cause mortality and major adverse cardiovascular event according to sleep duration. Kaplan–Meier unadjusted curves for all-cause mortality (**A**) and major adverse cardiovascular event (**B**) stratified according to sleep duration.

During a median follow-up of 186 months (interquartile range, 97–190), 1,095 deaths (11.4%) and 811 MACE (8.4%) were observed. Cumulative incidence curves showed a significantly higher risk of all-cause mortality and MACE in participants with > 8 h of sleep compared to other groups (both log-rank *p* < 0.001; Fig. 2). After adjusting for clinically relevant variables, participants with > 8 h of sleep had a significantly higher risk of all-cause mortality compared to those with 7–8 h of sleep (adjusted HR, 1.27; 95% CI, 1.04–1.54; *p* = 0.017). Participants with < 7 h of sleep showed a non-significant trend toward increased mortality (adjusted HR, 1.11; 95% CI, 0.97–1.27; *p* = 0.121). Similarly, those with irregular sleep also exhibited a modest but non-significant increase in mortality risk compared with their regular counterparts (adjusted HR, 1.10; 95% CI, 0.96–1.27; *p* = 0.164). In contrast, insufficient sleep was not associated with an increased risk of all-cause mortality (adjusted HR, 0.98; 95% CI, 0.85–1.13; *p* = 0.757; Table 2). MACE showed a similar trend, with a higher unadjusted incidence in the > 8 h group; however, none of the sleep duration, regularity, and sufficiency variables were significantly associated with MACE after adjustment (Supplementary Table 2).

**Table 2:** Incidence and HR of all-cause mortality according to self-reported sleep duration and quality.

	Incidence (%)	Unadjusted HR (95% CI)	*P*-value	Adjusted HR (95% CI)	*P*-value
< 7 h	404/4050 (10.0%)	0.89 (0.79–1.02)	0.086	1.11 (0.97–1.27)	0.121
7–8 h	545/4811 (11.3%)	Reference		Reference	
> 8 h	146/780 (18.7%)	1.74 (1.45–2.09)	< 0.001	1.27 (1.04–1.54)	0.017
Regular sleep time	796/7225 (11.0%)	Reference		Reference	
Irregular sleep time	299/2416 (12.4%)	1.16 (1.01–1.32)	0.031	1.10 (0.96–1.27)	0.164
Sufficient sleep time	803/6235 (12.9%)	Reference		Reference	
Insufficient sleep time	292/3406 (8.6%)	0.66 (0.58–0.76)	< 0.001	0.98 (0.85–1.13)	0.757

HR, hazard ratio; CI, confidence interval. Other abbreviations as in Table 1.

### Combined effects of sleep duration and quality on clinical outcomes

Combined analysis of sleep duration and regularity revealed that the highest risk of all-cause mortality was observed in participants with < 7 h and irregular sleep (adjusted HR, 1.28; 95% CI, 1.04–1.58; *p* = 0.020). Participants with > 8 h and regular sleep also showed a significantly increased mortality risk compared to those with 7–8 h of regular sleep (adjusted HR, 1.26; 95% CI, 1.01–1.58; *p* = 0.043; Table 3). No combination of sleep duration and regularity showed a statistically significant association with MACEs after adjustment. However, participants with long and irregular sleep showed a trend toward increased MACE risk (adjusted HR, 1.34; 95% CI, 0.88–2.05).

No significant associations were found in the combined analysis of sleep duration and sufficiency, although a trend toward increased mortality was observed in participants with > 8 h and insufficient sleep (adjusted HR, 1.52; 95% CI, 0.99–2.32; *p* = 0.053; Supplementary Table 3).

**Table 3:** Combined effects of sleep duration and regularity on all-cause mortality and MACE.

	< 7 h	7–8 h	> 8 h
**All-cause mortality**			
Unadjusted HR			
Regular sleep time	0.85 (0.73–0.99), *p* = 0.035	reference	1.85 (1.50–2.29), *p* < 0.001
Irregular sleep time	1.13 (0.92–1.38), *p* = 0.255	1.10 (0.91–1.33), *p* = 0.319	1.61 (1.15–2.25), *p* = 0.006
Adjusted HR			
Regular sleep time	1.06 (0.91–1.25), *p* = 0.451	reference	1.26 (1.01–1.58), *p* = 0.043
Irregular sleep time	1.28 (1.04–1.58), *p* = 0.020	1.04 (0.85–1.27), *p* = 0.718	1.33 (0.94–1.88), *p* = 0.107
**MACE**			
Unadjusted HR			
Regular sleep time	0.89 (0.75–1.06), *p* = 0.194	reference	1.54 (1.18–2.01), *p* = 0.001
Irregular sleep time	1.13 (0.89–1.43), *p* = 0.315	1.13 (0.91–1.41), *p* = 0.278	1.49 (0.99–2.25), *p* = 0.054
Adjusted HR			
Regular sleep time	1.02 (0.85–1.22), *p* = 0.844	reference	1.07 (0.80–1.42), *p* = 0.663
Irregular sleep time	1.17 (0.91–1.49), *p* = 0.222	1.03 (0.82–1.30), *p* = 0.806	1.34 (0.88–2.05), *p* = 0.172

HR, hazard ratio; CI, confidence interval; MACE, major adverse cardiovascular event; other abbreviations as listed in Table 1.

MACE is defined as a composite of cardiovascular death, spontaneous myocardial infarction, or stroke.

### Sex-and age-specific associations of sleep duration and sleep quality with clinical outcomes

**Fig. 3:**
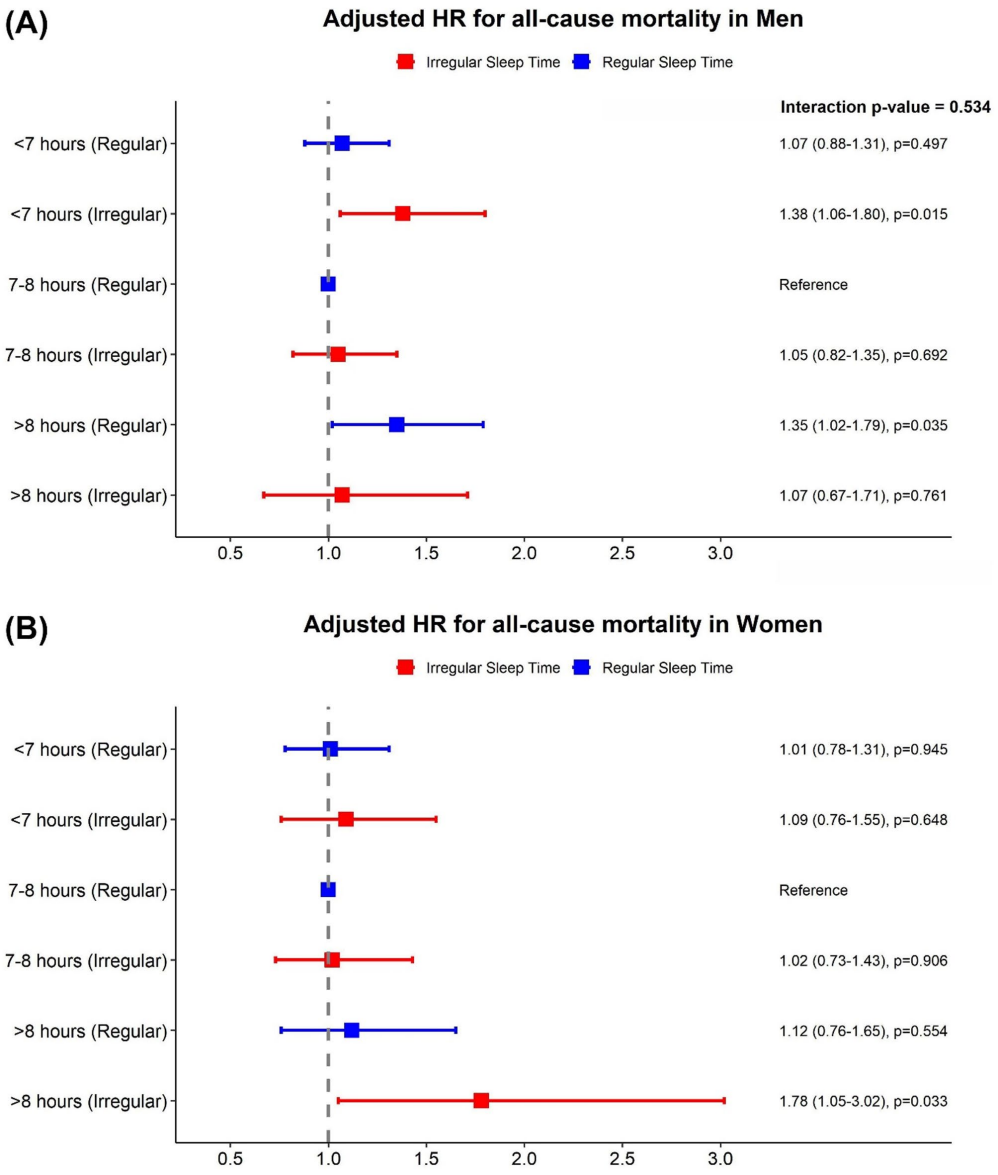
Adjusted hazard ratios for all-cause mortality by sleep duration and regularity, stratified by sex. Risk of all-cause mortality in men (**A**) and women (**B**).

**Fig. 4:**
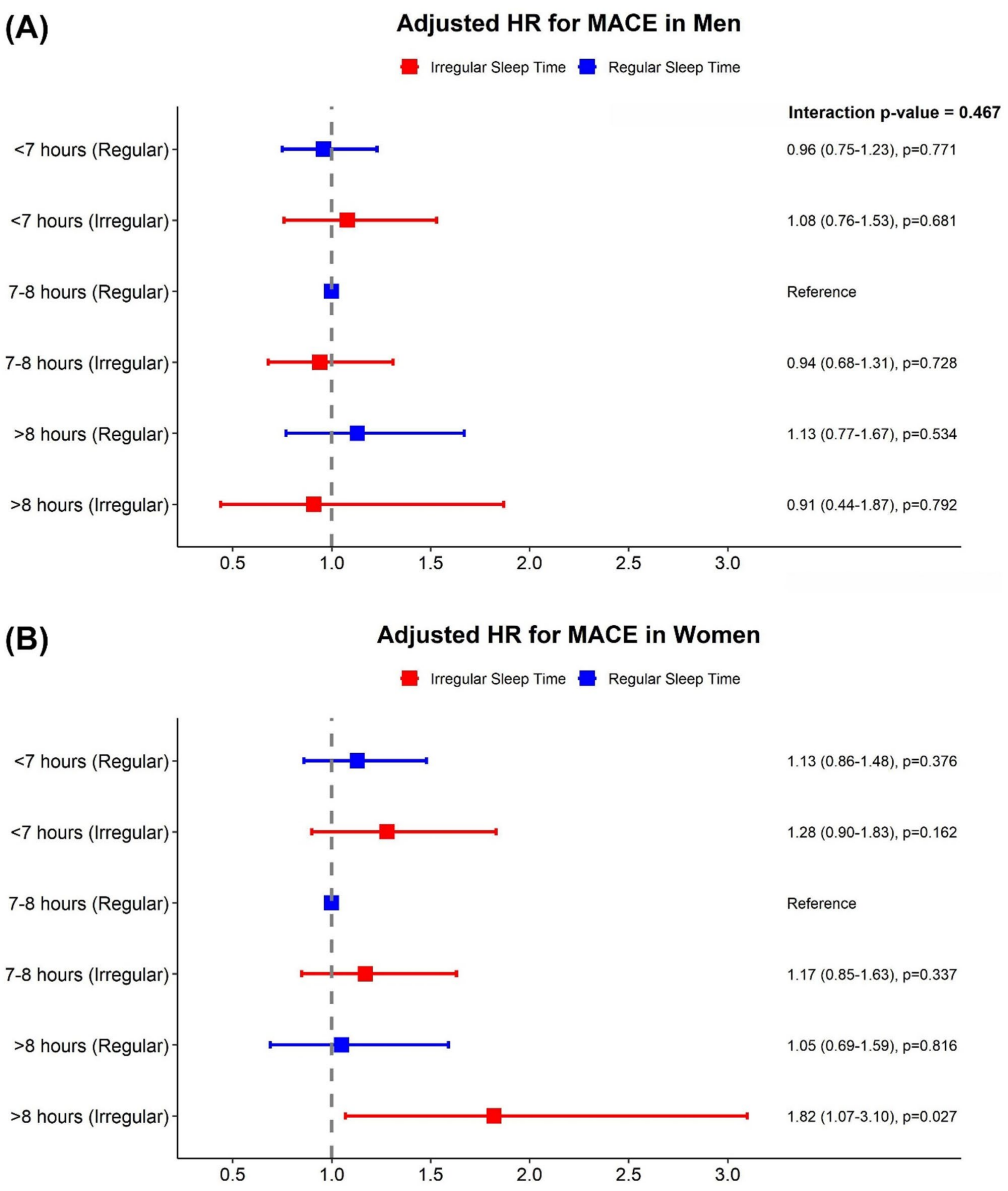
Adjusted hazard ratios for major adverse cardiovascular event by sleep duration and regularity, stratified by sex. Risk of major adverse cardiovascular event in men (**A**) and women (**B**).

In sex-stratified analyses, although the interaction terms between sex and sleep patterns were not statistically significant, some differences in associations were observed between men and women. Among men, two subgroups were associated with a higher risk of all-cause mortality compared to those with 7–8 h of regular sleep: participants with < 7 h and irregular sleep (adjusted HR, 1.38; 95% CI, 1.06–1.80, *p* = 0.015) and those with > 8 h and regular sleep (adjusted HR, 1.35; 95% CI, 1.02–1.79; *p* = 0.035). In women, the highest association for mortality was observed in those with > 8 h and irregular sleep (adjusted HR, 1.78; 95% CI, 1.05–3.02, *p* = 0.033; interaction, *p* = 0.534; Fig. 3). A similar sex-specific pattern was observed for MACE. While no significant associations were found in men, women with > 8 h and irregular sleep had a significant association with increased risk of MACE (adjusted HR, 1.82; 95% CI, 1.07–3.10, *p* = 0.027; interaction, *p* = 0.467; Fig. 4).

Additionally, age-stratified subgroup analyses showed no significant interactions by age, although some associations emerged within specific age groups (Supplementary Figs. 4–7). Participants aged 40–49 years with regular sleep of < 7 h had a significantly higher risk of MACE (adjusted HR, 1.46; 95% CI, 1.01–2.13; *p* = 0.047), and insufficient sleep regardless of sleep duration (adjusted HR, 1.74 and 1.81; *p* = 0.008 and 0.011). Among participants aged ≥ 60 years, increased all-cause mortality was observed in those with regular sleep duration > 8 h (adjusted HR, 1.35; 95% CI, 1.04–1.76; *p* = 0.025) and insufficient sleep with duration > 8 h (adjusted HR, 2.06; 95% CI, 1.26–3.37; *p* = 0.004).

## Discussion

In this large and extended follow-up after a period of > 15 years, robust associations were assessed even after comprehensive multivariable adjustment. The main findings of the current study were as follows. (1) Prolonged sleep duration (> 8 h) was independently associated with an increased risk of all-cause mortality compared with the reference group with 7–8 h of sleep. (2) Both prolonged sleep duration (> 8 h) with regular sleep and irregular sleep patterns combined with short sleep duration (< 7 h) were independently associated with an increased risk of all-cause mortality compared to the reference group (7–8 h with regular sleep). (3) In sex-stratified analyses, women with long and irregular sleep patterns and men with either long and regular or short and irregular sleep showed a significantly increased risk of all-cause mortality. (4) In age-stratified analyses, short sleep duration was generally associated with increased risk in participants aged 40–49 years, whereas prolonged sleep duration was associated with worse outcomes in those aged ≥ 60 years.

Cardiovascular diseases remain the leading cause of death worldwide, with modifiable risk factors playing crucial roles in disease progression and prevention^23^. While traditional risk factors such as hypertension, obesity, smoking, and sedentary lifestyles are well-documented^24^ the influence of sleep health is often underappreciated. Emerging epidemiological and mechanistic studies suggest that both insufficient and excessive sleep duration, poor sleep quality, and sleep disorders such as obstructive sleep apnea contribute to adverse cardiovascular outcomes ^1,3,8–11,25−34^. The bidirectional relationship between sleep disturbances and cardiovascular pathology highlights the need for a comprehensive approach to address sleep health in preventive and clinical care^8–11,35^. Furthermore, all-cause mortality, a broad indicator of population health, is also strongly linked to sleep patterns^8,9,11,25–28,30−34^. Our findings are consistent with previous studies. The present study also found that deviations from the 7–8-h sleep range were associated with increased mortality risk.

However, earlier studies focused primarily on sleep duration without incorporating the regularity or quality of sleep into their analytical framework^6,9–11,25,27,28,32,33,35^. We simultaneously evaluated sleep duration, sleep regularity, self-reported sleep sufficiency, and their combined effects on mortality and cardiovascular outcomes in a large, well-characterized, population-based cohort with long-term follow-up. Previous studies have reported increased mortality associated with short sleep duration^6,9–11,25,27,28,32,33,35^; however, the present study demonstrated that mortality was particularly elevated among individuals with short sleep durations accompanied by irregular sleep patterns. In contrast, long sleep duration showed an adverse association regardless of sleep regularity. Our findings established that irregular sleep patterns, particularly when combined with either short or long sleep durations, were associated with elevated mortality risk, highlighting the importance of sleep regularity as an independent risk factor. Although the underlying mechanisms remain unclear, these observations warrant further investigation to elucidate the causal pathways linking combined sleep patterns to adverse health outcomes.

Furthermore, we conducted sex-stratified analyses and revealed distinct patterns in men and women, where short irregular sleep duration was more detrimental to men, whereas long irregular sleep duration had a stronger association with mortality and MACE in women. The precise mechanisms underlying the observed sex-specific patterns remain incompletely understood, yet several plausible hypotheses warrant consideration. Hormonal regulation of sleep, including sex hormone effects, may contribute to differences in sleep-related outcomes between men and women. In women, hormonal transitions and greater exposure to psychosocial stress and caregiving responsibilities may impact sleep patterns^36^. In men, higher prevalence of sleep-disordered breathing, work-related stress such as long working hours may also play a role^37^. Additionally, short sleep duration was generally associated with increased risk in the younger age group, while long sleep duration was linked to worse outcomes in older adults^38^. This aligns with previous studies indicating stronger associations between long sleep and poor outcomes in older adults, although age- and sex-specific patterns remain underexplored. These findings underscore the potential value of incorporating sex- and age-specific considerations into sleep-related risk assessments and highlight the need for personalized approaches to sleep health in both clinical practice and public health strategies. Future research should further explore these mechanisms to better inform tailored interventions.

Taken together, these findings suggest that the optimal sleep duration may differ by demographic characteristics. While 7–8 h of sleep remains a broadly recommended range, our age-stratified results suggest that middle-aged individuals (particularly those aged 40–49 years) may be more vulnerable to short sleep duration, whereas older adults (≥ 60 years) appear to be more susceptible to the adverse effects of prolonged sleep. Similarly, sex-specific analyses indicate that men may be more affected by short and irregular sleep, whereas women are more vulnerable to long and irregular sleep patterns. These results support the development of individualized sleep health strategies that incorporate both sleep duration and regularity, tailored to age and sex.

In addition to clinical outcomes, the baseline characteristics across sleep duration groups revealed distinct sociodemographic patterns. Participants with prolonged sleep duration (> 8 h) were generally older, had a higher prevalence of comorbidities such as hypertension and diabetes, and were more likely to have lower education and income levels. Participants with short sleep duration (< 7 h) were younger, more likely to reside in urban areas, and had higher education and income levels. These findings suggest that sleep patterns may reflect not only the underlying health status but also broader lifestyle and social factors, which may help explain their differential associations with mortality risk. Although these comorbidities and socioeconomic factors were carefully adjusted, the possibility of residual confounding remained. Despite this, the existence of a direct causal relationship between sleep patterns and cardiovascular disease has not been conclusively established and requires further investigation.

Short sleep duration has well-known mechanisms linked to increased mortality. Sleep deprivation can lead to impaired glucose tolerance, elevated evening cortisol levels, heightened sympathetic nervous system activity, and reduced leptin secretion, potentially contributing to diabetes, hypertension, and obesity^38–41^. Thus, chronic sleep restriction can negatively influence overall health outcomes and increase the risk of chronic diseases and mortality.

However, the exact mechanisms linking increased self-reported sleep duration with higher mortality rates remain unclear. One proposed explanation is that prolonged self-reported sleep may reflect an individual’s increased need for sleep, which is indicative of reduced physiological reserves and a diminished ability to survive critical illnesses. Supporting this view, previous research has shown that individuals sleeping for approximately 7 h had a 10% increased risk of myocardial infarction compared to those sleeping for 8 h; however, the subsequent risk of mortality from myocardial infarction was 17% lower^42^. Additionally, prolonged sleep duration may indicate underlying undiagnosed health conditions or unmanaged comorbidities, such as obstructive sleep apnea, potentially elevating the risk of cardiovascular events and mortality^42,43^. Individuals initially experiencing insufficient sleep may extend their sleep duration as a compensatory response, eventually developing long sleep patterns associated with lower sleep efficiency, which is another factor linked to increased mortality risk.

Our study had several strengths. First, we used large-scale data with long-term follow-up to enhance the robustness of our findings. Second, our analysis comprehensively addressed overall sleep health by including not only sleep duration, but also sleep regularity and self-reported sufficiency. Third, we actively adjusted for numerous important factors, including key clinical variables (e.g., sex, BMI, hypertension, diabetes), detailed socioeconomic factors (e.g., income, education, and area of residence), and physical activity levels. These factors allowed us to thoroughly explore and control for differences in socioeconomic status across sleep patterns. Finally, our study highlighted notable sex-based differences in associations with poor sleep patterns, providing further insight into sleep-related health disparities.

This study has several limitations. First, given the observational nature of the study, we cannot infer the causality between sleep patterns and the risk of all-cause mortality or cardiovascular outcomes. Although we adjusted for a wide range of potential confounders, the possibility of residual confounders from unmeasured or imprecisely measured variables cannot be excluded. In particular, sleep disorders, such as obstructive sleep apnea, which may influence both sleep patterns and cardiovascular outcomes, were not objectively assessed in this study. This limitation could have led to the misclassification of sleep quality or underestimation of associated risks. Second, sleep duration and regularity were assessed using self-reported questionnaires, which are subject to recall bias and misclassification. Moreover, the assessment of sleep regularity did not distinguish between weekday and weekend patterns; thus, potential circadian misalignment could not be captured. Such non-differential misclassification is likely to bias the results toward null, potentially underestimating the true associations. Additionally, objective sleep measurements such as actigraphy or polysomnography were not available, limiting the precision of sleep pattern assessment. Third, we assessed baseline sleep characteristics only once without accounting for changes in sleep behavior over a long follow-up period. Repeated measurements may better capture the dynamic nature of sleep and its effects on health. Finally, although we adjusted for extensive socioeconomic factors, we did not fully assess other potential determinants of sleep duration such as intrinsic sleep needs, work schedules, psychological stress, and environmental influences, all of which could differentially impact health outcomes.

In conclusion, this large prospective cohort study found a complex association between sleep duration, regularity, and mortality. Long sleep durations were associated with an increased risk of mortality among individuals with regular sleep, whereas short sleep durations were associated with higher risk among those with irregular sleep. Although the interaction between sex and sleep patterns was not statistically significant, stratified analyses suggested potential sex-specific trends. These patterns may help inform tailored sleep health strategies that consider both sleep characteristics and sex.

## Supplementary Information

Below is the link to the electronic supplementary material.

Supplementary Material 1

## Data Availability

The datasets generated and/or analyzed during the current study are available from the corresponding author upon reasonable request.
