# Supplementary material for: The impact of sleep health on cardiovascular and all-cause mortality in the general population
Source: Sci Rep. 2025 Aug 16;15:30034. doi: 10.1038/s41598-025-15828-6 (PMC12357935; doi:10.1038/s41598-025-15828-6)

**Online Supplementary Appendix**

The appendix is provided by the authors to provide readers with additional information about their work. Supplement to: J Park et al. **“The impact of sleep health on cardiovascular and all-cause mortality in the general population”**

**Supplemental Tables**

Supplementary Table 1. Comparison of baseline characteristics between participants with and without follow-up completion

Supplementary Table 2. Baseline sleep characteristics according to sex

Supplementary Table 3. Incidence and HR for major adverse cardiovascular events according to self-reported sleep duration and quality

Supplementary Table 4. Combined effects of sleep duration and sufficiency on all-cause mortality and MACE

**Supplemental Figures**

Supplementary Figure 1. The distribution of sleep duration among study participants.

Supplementary Figure 2. Adjusted hazard ratios for all-cause mortality by sleep duration and sufficiency, stratified by sex.

Supplementary Figure 3. Adjusted hazard ratios for major adverse cardiovascular events according to sleep duration and sufficiency, stratified by sex.

Supplementary Figure 4. Adjusted hazard ratios for all-cause mortality by sleep duration and regularity, stratified by age group.

Supplementary Figure 5. Adjusted hazard ratios for major adverse cardiovascular events according to sleep duration and regularity, stratified by age group.

Supplementary Figure 6. Adjusted hazard ratios for all-cause mortality by sleep duration and sufficiency, stratified by age group.

Supplementary Figure 7. Adjusted hazard ratios for major adverse cardiovascular events according to sleep duration and sufficiency, stratified by age group.

**Supplementary Table 1**. Comparison of baseline characteristics between participants with and without follow-up completion

| Characteristic | Completed follow-up (N=6,954) | Lost to follow-up  (N=2,687) | P- value |
| --- | --- | --- | --- |
| Age, mean (SD), years | 52.48 ± 8.82 | 51.14 ± 8.99 | <0.001 |
| Male sex, N (%) | 3358 (48.3) | 1213 (45.1) | 0.006 |
| Area |  |  | <0.001 |
| Urban (Ansan) | 3189 (45.9) | 1692 (63.0) |  |
| Rural (Ansung) | 3765 (54.1) | 995 (37.0) |  |
| Body mass index, N (%), kg/m2 |  |  | 0.088 |
| <18.5 | 133 (1.9) | 37 (1.4) |  |
| 18.5–22.9 | 2025 (29.3) | 747 (28.0) |  |
| 23.0–24.9 | 1797 (26.0) | 715 (26.8) |  |
| 25.0–29.9 | 2621 (38.0) | 1013 (38.0) |  |
| ≥ 30.0 | 329 (4.8) | 152 (5.7) |  |
| Waist circumference, mean (SD), cm | 82.98 ± 8.72 | 82.78 ± 8.95 | 0.309 |
| Monthly income, N (%), *10^4^ KRW |  |  | <0.001 |
| Low (<100) | 2514 (36.7) | 831 (31.3) |  |
| Medium (100-199) | 1966 (28.7) | 817 (30.8) |  |
| High (≥200) | 2362 (34.5) | 1005 (37.9) |  |
| Education, N (%) |  |  | 0.001 |
| Lower than middle school | 2359 (34.1) | 816 (30.5) |  |
| Middle school | 1604 (23.2) | 609 (22.8) |  |
| High school | 2068 (29.9) | 855 (32.0) |  |
| University and college | 881 (12.7) | 396 (14.8) |  |
| Smoking status, N (%) |  |  | 0.024 |
| Current-smoker | 1731 (25.2) | 723 (27.1) |  |
| Ex-smoker | 1100 (16.0) | 375 (14.1) |  |
| Never-smoker | 4049 (58.9) | 1565 (58.8) |  |
| Alcohol drinking, N (%) |  |  | 0.456 |
| Current-drinker | 3278 (47.4) | 1262 (47.2) |  |
| Ex-drinker | 463 (6.7) | 162 (6.1) |  |
| Never-drinker | 3169 (45.9) | 1250 (46.7) |  |
| Physical acitivity, N (%), METs-hour/week |  |  | <0.001 |
| <7.5 | 498 (7.2) | 249 (9.3) |  |
| 7.5-14.9 | 1267 (18.2) | 548 (20.4) |  |
| 15.0-30.0 | 1975 (28.4) | 935 (34.8) |  |
| ≥30.0 | 3214 (46.2) | 955 (35.5) |  |
| Systolic blood pressure, mean (SD), mmHg | 124.53 ± 18.67 | 124.46 ± 19.14 | 0.883 |
| Diastolic blood pressure, mean (SD), mmHg | 81.64 ± 11.59 | 81.69 ± 12.28 | 0.853 |
| Medical history, N (%) |  |  |  |
| Hypertension | 2579 (37.1) | 1009 (37.6) | 0.694 |
| Diabetes mellitus | 784 (11.3) | 276 (10.3) | 0.172 |
| Dyslipidemia | 2873 (41.4) | 1116 (41.6) | 0.891 |
| Chronic kidney disease | 136 (2.0) | 63 (2.3) | 0.262 |
| Coronary artery disease | 50 (0.7) | 20 (0.7) | 1 |
| Heart failure | 13 (0.2) | 4 (0.1) | 0.897 |
| Laboratory data, mean (SD) |  |  |  |
| eGFR, mL/min/1.73m2 | 92.16 ± 14.09 | 92.00 ± 14.68 | 0.617 |
| Fasting glucose, mg/dL | 92.17 ± 22.17 | 92.98 ± 24.07 | 0.023 ^a^ |
| Hemoglobin a1c, % | 5.80 ± 0.93 | 5.78 ± 0.92 | 0.363 ^a^ |
| Total cholesterol, mg/dL | 197.14 ± 36.30 | 202.09 ± 37.77 | <0.001 |
| Triglyceride, mg/dL | 152.29 ± 105.83 | 154.65 ± 118.12 | 0.534 ^a^ |
| HDL cholesterol, mg/dL | 49.47 ± 11.93 | 49.82 ± 11.70 | 0.201 |
| LDL cholesterol, mg/dL | 121.25 ± 31.70 | 125.18 ± 32.68 | <0.001 |
| Self-reported sleep quality, N (%) |  |  |  |
| Sufficient sleep duration | 4560 (65.6) | 1675 (62.3) | 0.003 |
| Regular sleep time | 5221 (75.1) | 2004 (74.6) | 0.632 |

Values are mean ± standard deviation or n (%) unless indicated otherwise. Percentages may not total 100% because of rounding

Glomerular filtration rate was calculated using CKD-EPI equations.

^a^ Assessed by nonparametric test

eGFR, Estimated Glomerular Filtration Rate; HDL, High-Density Lipoprotein; KRW, Korean Won; LDL, Low-Density Lipoprotein; METs, Metabolic Equivalents; SD, Standard Deviation

**Supplementary Table 2**. Baseline sleep characteristics according to sex

|  | Men  (N=4,571) | Women  (N=5070) | *P*-value |
| --- | --- | --- | --- |
| Sleep duration, hours/day | 6.83 ± 1.32 | 6.68 (1.43) | <0.001 |
| <7 hours | 1785 (39.1) | 2265 (44.7) |  |
| 7–8 hours | 2422 (53.0) | 2389 (47.1) |  |
| >8 hours | 364 (8.0) | 416 (8.2) | <0.001 |
| Self-reported sleep quality, N (%) |  |  |  |
| Sufficient sleep duration | 3127 (68.4) | 3108 (61.3) | <0.001 |
| Regular sleep time | 3429 (75.0) | 3796 (74.9) | 0.889 |

Values are presented as mean ± standard deviation or n (%).

**Supplementary Table 3.** Incidence and HR for major adverse cardiovascular events according to self-reported sleep duration and quality

|  | Incidence (%) | Unadjusted HR  (95% CI) | *P*-value | Adjusted HR  (95% CI) | *P*-value |
| --- | --- | --- | --- | --- | --- |
| <7 hours | 312/4050 (7.7%) | 0.92 (0.79–1.07) | 0.262 | 1.05 (0.90–1.23) | 0.534 |
| 7–8 hours | 407/4811 (8.5%) | reference |  | reference |  |
| >8 hours | 92/780 (11.8%) | 1.48 (1.18–1.86) | 0.001 | 1.12 (0.88–1.43) | 0.347 |
|  |  |  |  |  |  |
| Regular sleep time | 590/7225 (8.2%) | reference |  | reference |  |
| Irregular sleep time | 221/2416 (9.1%) | 1.17 (1.01–1.37) | 0.048 | 1.10 (0.93–1.29) | 0.268 |
|  |  |  |  |  |  |
| Sufficient sleep time | 566/6235 (9.1%) | reference |  | reference |  |
| Insufficient sleep time | 245/3406 (7.2%) | 0.78 (0.67–0.91) | 0.001 | 1.08 (0.92–1.26) | 0.355 |

HR, hazard ratio; CI, confidence interval; MACE, major adverse cardiovascular event; other abbreviations as listed in Table 1.

MACE is defined as a composite of cardiovascular death, spontaneous myocardial infarction, or stroke.

**Supplementary Table 4.** Combined effects of sleep duration and sufficiency on all-cause mortality and MACE

| All-cause mortality | | | |
| --- | --- | --- | --- |
| Unadjusted HR | **<7 hours** | **7–8 hours** | **>8 hours** |
| Sufficient sleep time | 0.94 (0.80–1.10), p=0.449 | reference | 1.59 (1.30–1.94), p<0.001 |
| Insufficient sleep time | 0.68 (0.57–0.81), p<0.001 | 0.61 (0.49–0.77), p<0.001 | 1.48 (0.99–2.22), p=0.056 |
| Adjusted HR | **<7 hours** | **7–8 hours** | **>8 hours** |
| Sufficient sleep time | 1.12 (0.95–1.32), p=0.175 | reference | 1.20 (0.97–1.49), p=0.088 |
| Insufficient sleep time | 1.06 (0.88–1.28), p=0.525 | 0.92 (0.73–1.16), p=0.474 | 1.52 (0.99–2.32), p=0.053 |
| MACE | | | |
| Unadjusted HR | **<7 hours** | **7–8 hours** | **>8 hours** |
| Sufficient sleep time | 1.01 (0.84–1.21), p=0.920 | reference | 1.43 (1.11–1.84), p=0.005 |
| Insufficient sleep time | 0.76 (0.63–0.93), p=0.008 | 0.85 (0.67–1.07), p=0.164 | 1.39 (0.84–2.29), p=0.203 |
| Adjusted HR | **<7 hours** | **7–8 hours** | **>8 hours** |
| Sufficient sleep time | 1.09 (0.90–1.32), p=0.391 | reference | 1.16 (0.89–1.51), p=0.272 |
| Insufficient sleep time | 1.09 (0.88–1.34), p=0.420 | 1.18 (0.92–1.5), p=0.185 | 1.17 (0.68–2.00), p=0.572 |

HR, hazard ratio; CI, confidence interval; MACE, major adverse cardiovascular event; other abbreviations as listed in Table 1.

MACE is defined as a composite of cardiovascular death, spontaneous myocardial infarction, or stroke.

**Supplementary Figure 1.** The distribution of sleep duration among study participants.

**
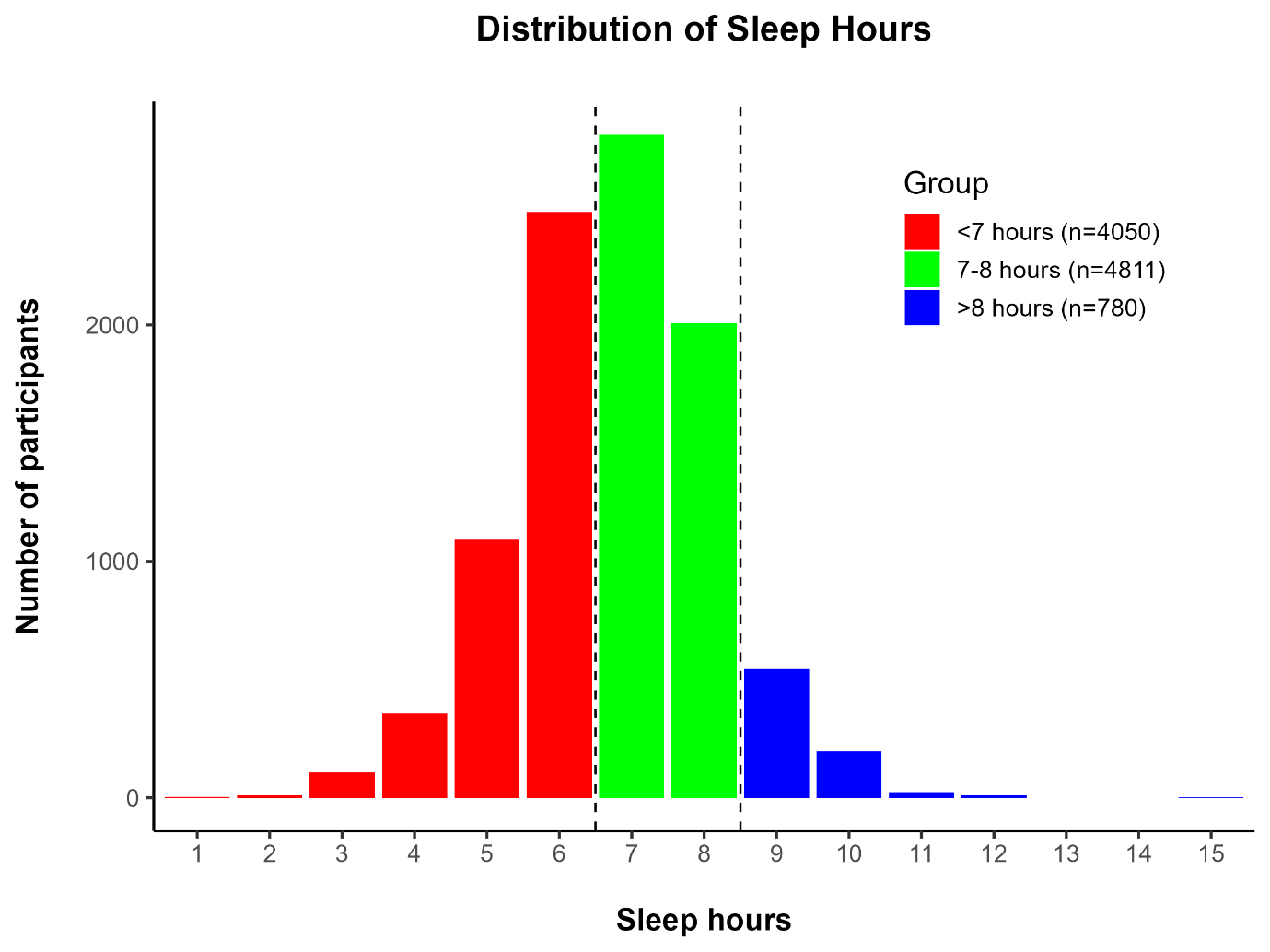
**

**Supplementary Figure 2.** Adjusted hazard ratios for all-cause mortality by sleep duration and sufficiency, stratified by sex. Risk of all-cause mortality in men (A) and women (B).


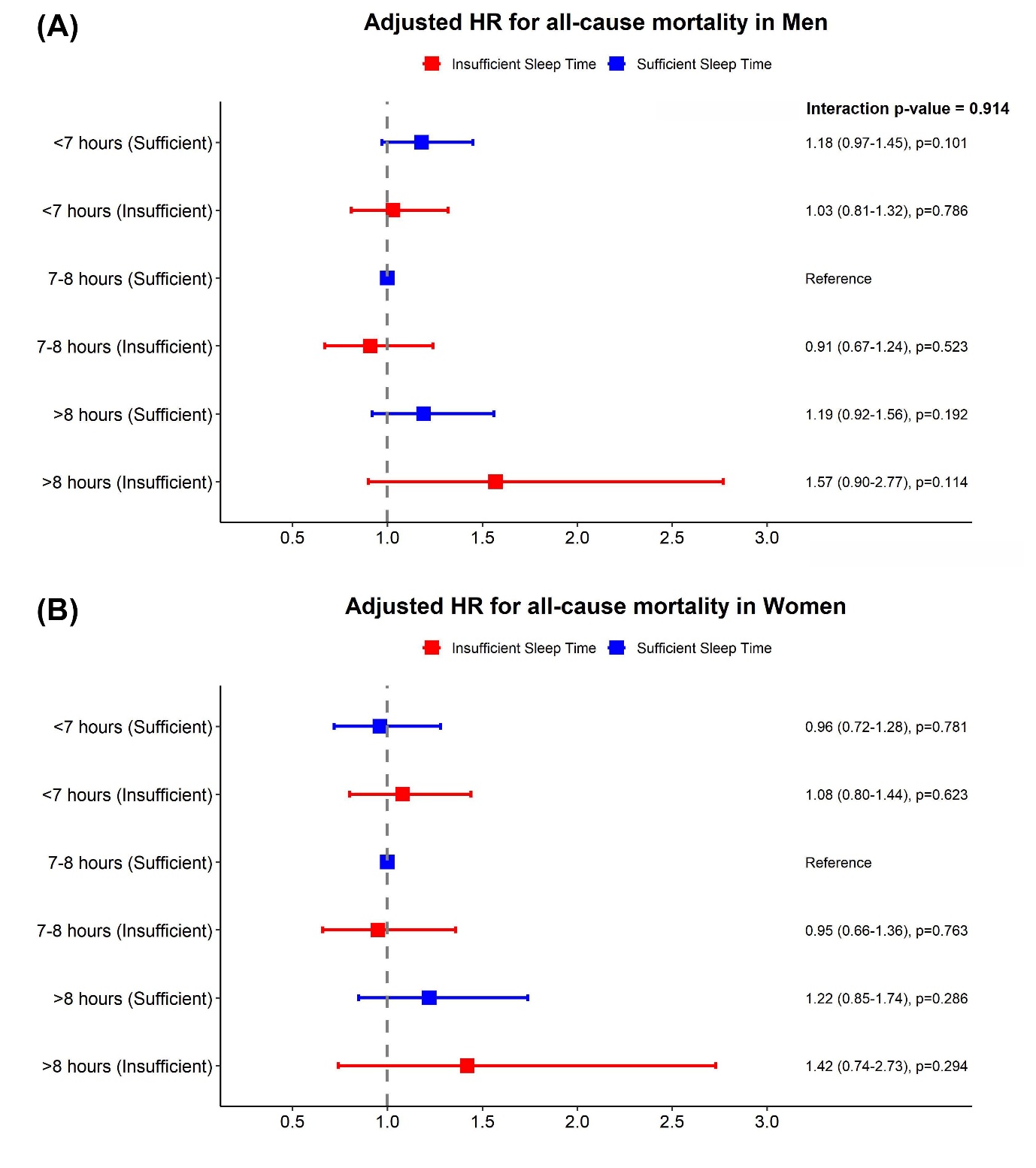


**Supplementary Figure 3.** Adjusted hazard ratios for major adverse cardiovascular events according to sleep duration and sufficiency, stratified by sex. Risk of major adverse cardiovascular event in men (A) and women (B).


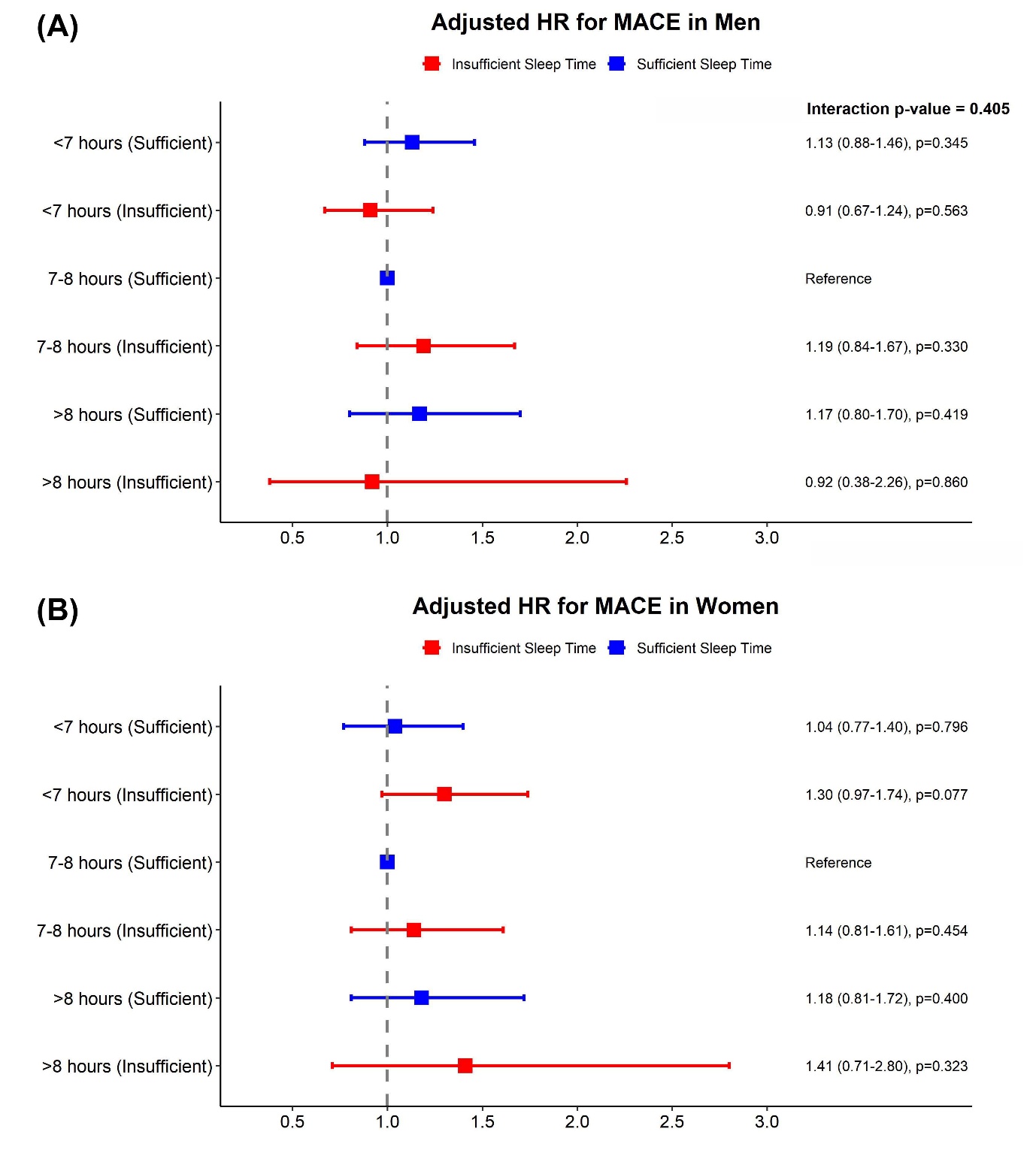


Supplementary Figure 4. Adjusted hazard ratios for all-cause mortality by sleep duration and regularity, stratified by age group. Risk of all-cause mortality in aged 40–49 years (A), aged 50–59 years (B), and ≥60 years (C).


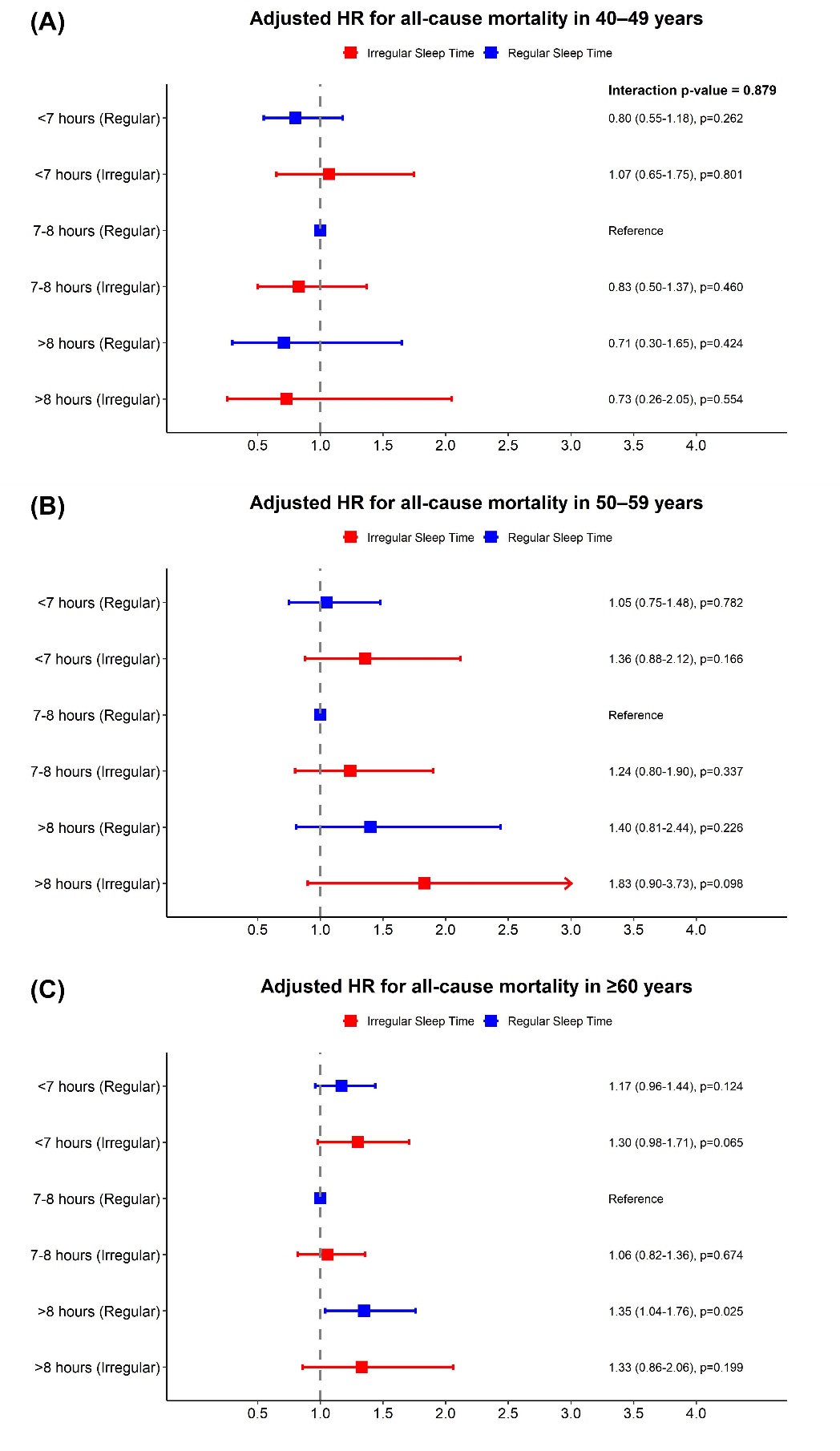


**Supplementary Figure 5.** Adjusted hazard ratios for major adverse cardiovascular events according to sleep duration and regularity, stratified by age group. Risk of major adverse cardiovascular event in aged 40–49 years (A), aged 50–59 years (B), and ≥60 years (C).


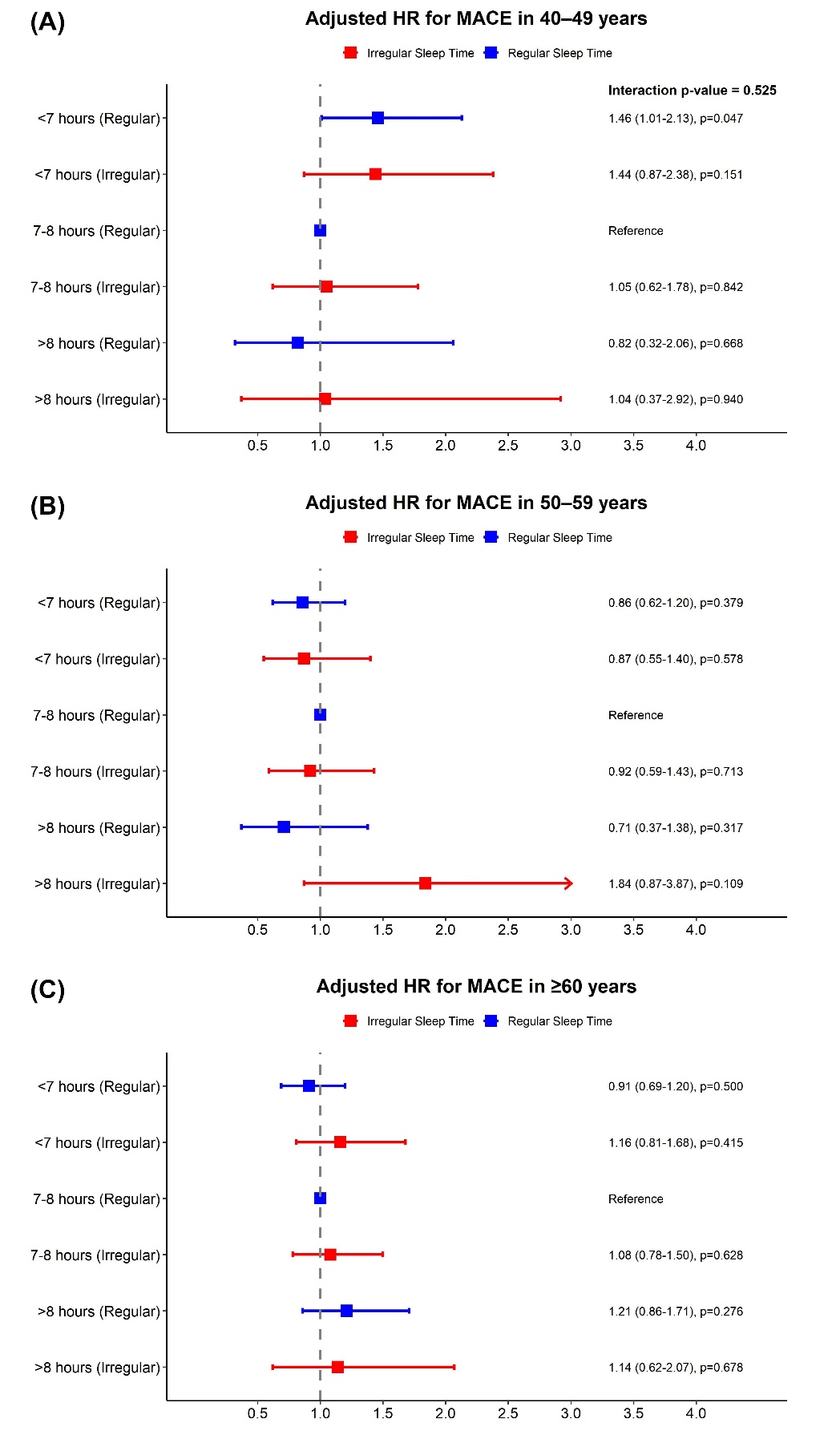


**Supplementary Figure 6.** Adjusted hazard ratios for all-cause mortality by sleep duration and sufficiency, stratified by age group. Risk of all-cause mortality in aged 40–49 years (A), aged 50–59 years (B), and ≥60 years (C).


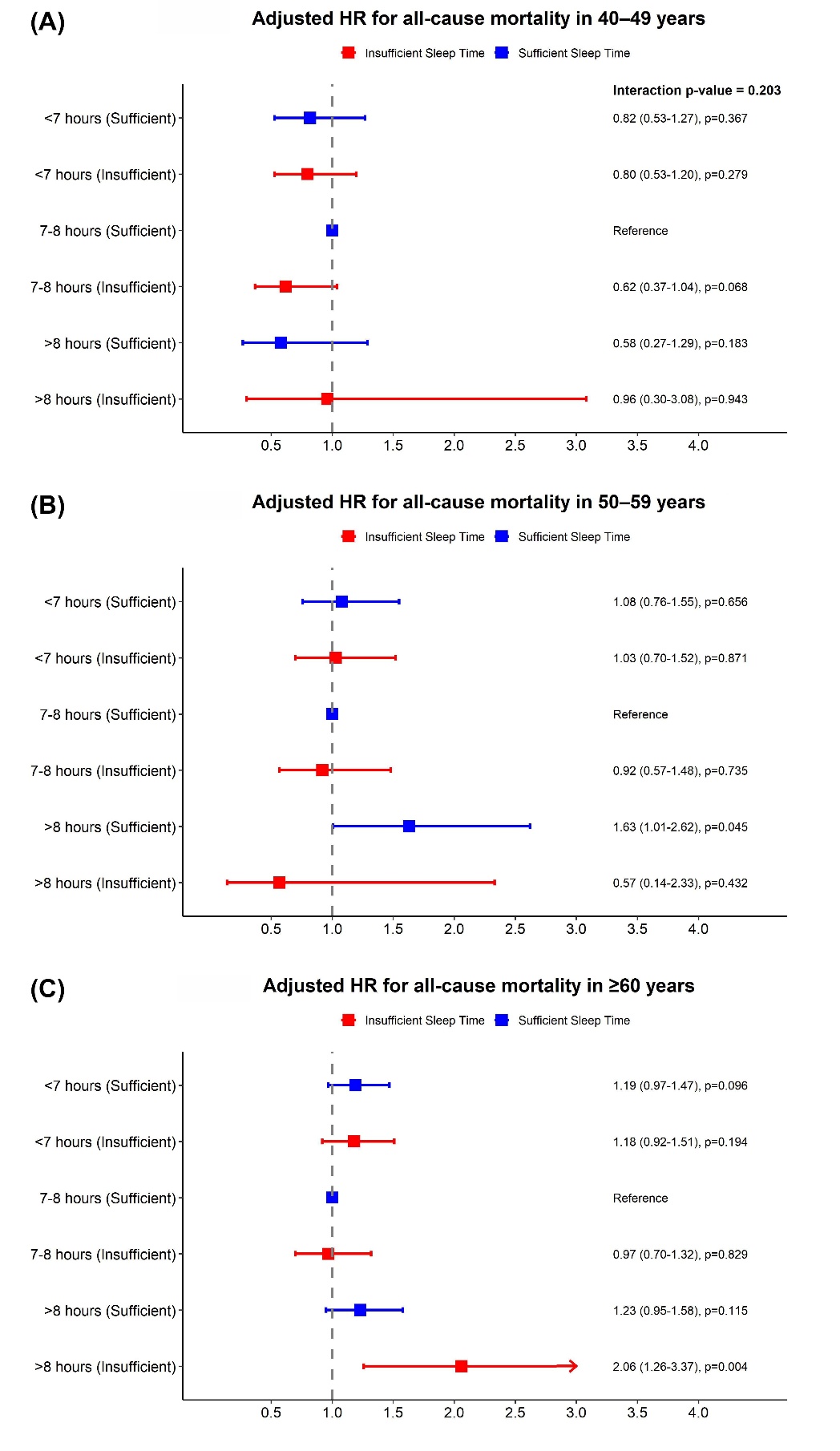


**Supplementary Figure 7.** Adjusted hazard ratios for major adverse cardiovascular events according to sleep duration and sufficiency, stratified by age group. Risk of major adverse cardiovascular event in aged 40–49 years (A), aged 50–59 years (B), and ≥60 years (C).


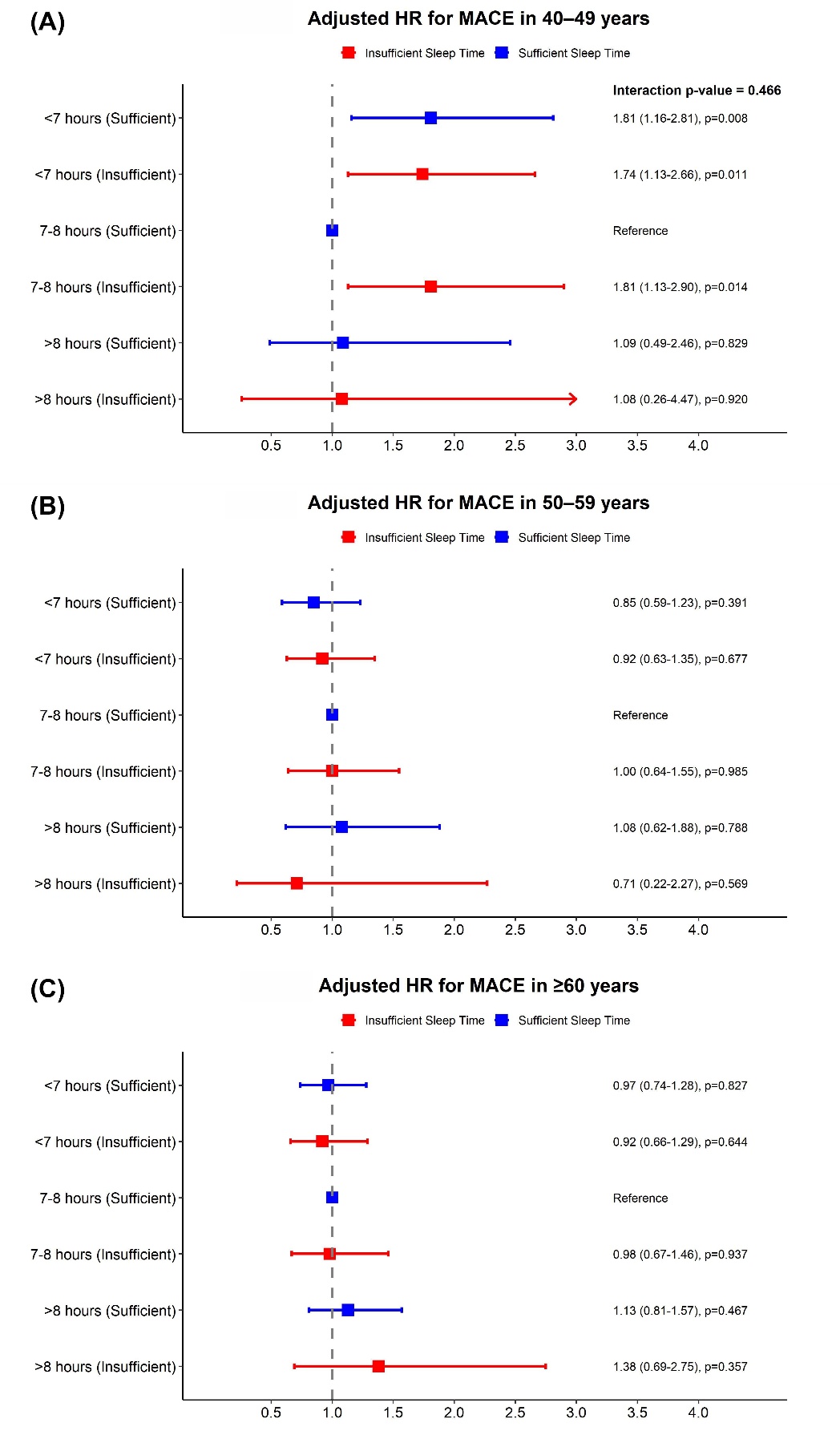

Supplement: Supplementary file 1 — Supplementary Material 1 [file 41598_2025_15828_MOESM1_ESM.docx]
